# Estimated Worldwide Mortality Attributed to Secondhand Tobacco Smoke Exposure, 1990-2016

**DOI:** 10.1001/jamanetworkopen.2020.1177

**Published:** 2020-03-17

**Authors:** Hamza Yousuf, Martijn Hofstra, Jan Tijssen, Brian Leenen, Jan Willem Lindemans, Albert van Rossum, Jagat Narula, Leonard Hofstra

**Affiliations:** 1 Department of Cardiology, VU University Medical Center, Amsterdam University Medical Center, Amsterdam, the Netherlands; 2 Erasmus University, Rotterdam, the Netherlands; 3 Department of Cardiology, Amsterdam Medical Center, Amsterdam University Medical Center, Amsterdam, the Netherlands; 4 Center for Advanced Hindsight, Duke University, Durham, North Carolina; 5 Department of Cardiology, Mount Sinai Hospital, New York City, New York

## Abstract

**Importance:**

The World Health Organization estimates that the 1 billion individuals who smoke worldwide contribute to the 880 000 secondhand smoke (SHS)–related deaths among individuals who do not smoke each year. A better understanding of the scale of harm of SHS to those who do not smoke could increase awareness of the consequences of smoking and help to design measures to protect individuals who do not smoke, especially children.

**Objective:**

To calculate the number of individuals who smoke associated with the death of 1 individual who died of SHS exposure both on a global scale and in various World Bank regions.

**Design, Setting, and Participants:**

In this cross-sectional epidemiologic assessment, data from Our World in Data were used to tabulate the number of individuals who smoke in each country and number of premature deaths related to SHS in that country from 1990 to 2016. The mean number of cigarettes consumed in all countries was also included in analyses. Data were collected for the following World Bank regions: North America, Latin America and the Caribbean, Europe and Central Asia, the Middle East and North Africa, sub-Saharan Africa, South Asia, and East Asia and the Pacific from 1990 and 2016. Statistical analysis was conducted in July 2019.

**Exposure:**

Secondhand smoke.

**Main Outcomes and Measures:**

The pack-year index, calculated as the number of pack-years associated with the death of 1 individual who does not smoke but was exposed to SHS, and the SHS index, calculated as the number of individuals who smoked for 24 years (ie, the mean duration of smoking) associated with the death of 1 individual who does not smoke.

**Results:**

Globally, the SHS index changed favorably, from 31.3 (95% CI, 30.6-32.0) individuals who smoked associated with the death of 1 individual who did not smoke in 1990 to 52.3 (95% CI, 51.2-53.5) individuals who smoked in 2016. There was a wide regional variation in the 2016 secondhand smoke index, from 42.6 (95% CI, 41.6-43.5) individuals who smoked in the Middle East and North Africa to 85.7 (95% CI, 83.8-87.7) individuals who smoked in North America. Worldwide, the pack-year index also changed favorably from 751.9 (95% CI, 736.3-770.7) pack-years associated with 1 death in 1990 to 1255.9 (95% CI, 1227.2-1284.4) pack-years in 2016.

**Conclusions and Relevance:**

In this study, the substantial disparity among regions in both the SHS index and pack-year index reflected large differences in the scale of the harm of SHS on those who do not smoke. This information may help local policy makers implement measures to better protect those who do not smoke and increase public engagement. Although the number of pack-years and the number of individuals who smoke associated with the death of 1 individual who did not smoke favorably changed over the study period, as of 2016, 52.3 individuals who smoked were associated with the death of 1 individual who did not smoke.

## Key Points

**Question:**

How many individuals who smoke are associated with the death of 1 individual who does not smoke but was exposed to secondhand smoke?

**Findings:**

This cross-sectional epidemiologic assessment used Our World in Data to calculate how many individuals who smoked for a mean of 24 years were associated with the death of 1 individual who died of exposure to secondhand smoke; globally, this changed from 31.3 individuals who smoked in 1990 to 52.3 individuals who smoked in 2016.

**Meaning:**

The findings of this study could help policy makers better understand the scale of harm associated with secondhand smoke and improve awareness in the general public.

## Teaser

This cross-sectional epidemiologic assessment calculates the number of individuals who smoke associated with the death of 1 individual who does not smoke and assesses trends in this number from 1990 to 2016.

## Introduction

Tobacco use is the number 1 preventable cause of death worldwide, contributing to 6 million deaths every year.^1^ However, the deleterious associations of tobacco smoking with health are not limited to those who smoke, but they also affect those in the vicinity who do not smoke, ie, those exposed to secondhand smoke (SHS). Compelling correlative evidence claims that exposure to SHS is responsible for the death of more than 880 000 individuals worldwide every year.^2,3^ Compared with children with no SHS exposure, children exposed to SHS are at an increased risk of sudden infant death syndrome, acute respiratory infections, aural dysfunction, and the aggravation of asthma.^4,5^ Even a low-dose SHS exposure has been demonstrated to be associated with platelet activation and endothelial dysfunction and to be associated with deleterious effects on the cardiovascular system.^6^ Exposure to SHS has also been associated with more extensive coronary artery disease and a 20% to 30% increase in cardiovascular events compared with individuals with no exposure.^6^ A similar increase in lung cancer is reported among individuals exposed to SHS compared with those not exposed.^7^ It is also intriguing to review the beneficial associations of restricting SHS exposure. Globally, as much as a 15% lower rate of myocardial infarction was reported widely after the first year of enforcing smoking bans.^8^ Smoking bans in bars and restaurants have been introduced in most countries and have demonstrated health-related benefits.^8^ For instance, a smoking ban reduced admissions for asthmatic emergencies among children by 11 000 annually in Scotland alone.^9^ Although it is widely believed that SHS is associated with extensive harm and the number of individuals who smoke by country and globally has been reported alongside the number of individuals who have died of SHS exposure, the scale of the consequences of smoking on those who do not smoke has not been developed.

The aim of the current study was to better understand the scale of the harm caused by SHS to those who do not smoke on a global scale and in different regions. For this purpose, we calculated the SHS index (SHSI), which measures the number of individuals who smoke associated with the death of 1 individual who does not. We also calculated the pack-year index (PYI), which measures the number of pack-years of smoking associated with the death of 1 individual who does not smoke.

## Methods

Our report follows the Strengthening the Reporting of Observational Studies in Epidemiology (STROBE) reporting guideline. Ethical approval for our investigation was provided by the medical ethics committee of the VU University Medical Center. Given that this study was based on preexisting, open access data sets, informed consent was neither required nor possible.

### Data Transformation and Source

Our primary data source was Our World in Data (OWID), developed by researchers at the Oxford Martin Program on Global Development of the University of Oxford and funded by the Bill and Melinda Gates Foundation.^3^ The OWID database comprises a combination and interpretation of numerous existing high-quality data sets, including the World Health Organization Global Health Observatory Data Repository,^10^ the International Mortality and Smoking Statistics,^11^ and the Institute of Health Metrics and Evaluation Global Burden of Disease database.^12^

### Number of Individuals Who Smoke

The number of individuals who actively smoke was obtained from OWID*.* Most data were available from 1990 until 2016. Some data sets only contained data points until 2012, and we extrapolated the data to 2016 (eAppendix in the Supplement).

### Individuals Who Died of SHS Exposure

An individual who died of SHS exposure, as defined by OWID, is a person who did not actively smoke but was exposed to smoke and died of smoke-related illnesses. Secondhand tobacco smoking is also called passive or sidestream smoking. The annual number of individuals who died of SHS exposure is available from the OWID database. In addition, the Global Burden of Disease report has used the definition from a meta-analysis of 19 studies,^2^ largely based on comparative risk assessment approaches in which the proportion of disease-specific burden associated with SHS exposure was calculated. These data provide the extent of exposure to SHS, the effect size of SHS exposure, and the level of risk associated with SHS exposure. To provide the best estimate of the scale of SHS harm, the number of individuals who died of SHS exposure was divided by the total world population.^3^

### Time From SHS Exposure to Onset of Disease

We needed to explore the association of the number of individuals who died of SHS exposure in a given year with the number of individuals who actively smoked in preceding years. To better understand this association given variable delay to the harm, we calculated the numbers of individuals who died of SHS exposure with variation in lead-time delay of 0, 2, 10, and 20 years.

### PYI to Measure Number of Pack-Years Associated With the Death of 1 Individual Who Does Not Smoke

To differentiate between the harm associated with light and heavy smoking, the number of pack-years associated with the death 1 individual who does not smoke was calculated. The number of cigarettes smoked per day is an essential factor for the calculation of the number of pack-years associated with harm (ie, the PYI) as well as the number of individuals who actively smoke and were associated with harm (ie, the SHSI). We defined 1 pack-year as 1 year of smoking a pack of cigarettes each day. The OWID data set on global smoking behavior provided the estimated number of cigarettes smoked per day and also estimated lower and upper bounds.^13^ To calculate the number needed to harm, the number of individuals who died of SHS exposure was divided by the total number of individuals who were actively smoking that year. The proportion of cigarettes consumed per day per person divided by the number of cigarettes contained in 1 pack was used to correct the differences in daily cigarette consumption across time and regions. Because the data on individuals who died of SHS exposure were only available in clusters of 5 years, linear interpolation was conducted to determine the intermediate numbers of individuals in this group. The year for which the number of individuals who actively smoked and the number of cigarettes smoked per day was calculated was linked to the number of individuals who died of SHS exposure in a later year, based on 4 lead-time delays (ie, 0, 2, 10, and 20 years).

The global number of pack-years in a given year was based on the total number of individuals who smoked worldwide in that year multiplied by the mean number of cigarettes smoked, normalized for a pack of 20 cigarettes. These data were extracted from OWID. The following equation was formulated:
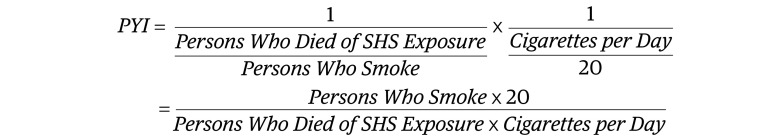
For the calculation of the PYI, we used 0-, 2-, 10- and 20-year delays between the number of individuals who smoked and the number of individuals who died of SHS exposure.

### SHSI to Measure Number of Individuals Who Smoked Associated With the Death of 1 Individual Who Did Not

The SHSI was calculated from the PYI (ie, pack-years needed to harm), normalized for the lifetime years of individuals who smoke daily. To derive the number of individuals who smoke associated with the death of 1 individual who does not, we defined mean smoking duration. It has been reported that 90% of individuals who smoke daily begin before the age of 25 years and that the mean duration of daily smoking is 24 years.^14^

Data on cigarette consumption were obtained from OWID. Mean daily cigarette consumption varied significantly across time, regions, and countries. To correct for these variations, mean daily cigarette consumption was taken into account for every country for the relevant years in the calculation of the number needed to harm.^15^

The calculation of the SHSI was based on the total number of individuals who smoked worldwide in a given year multiplied by the mean number of cigarettes smoked (normalized for a pack of 20 cigarettes) and divided by the mean smoking time span of 24 years. In applying this approach, the following equation was formulated:
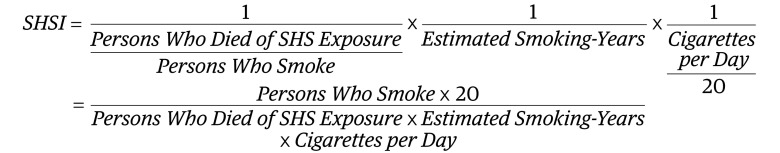
We used 0-, 2-, 10-, and 20-year delays between the number of individuals who smoked and the number of individuals who died of SHS exposure.

### Calculation of the SHSI for Geographic Regions 

The SHSI was calculated for all World Bank regions, including South Asia, North America, the Middle East and North Africa, East Asia and the Pacific, Europe and Central Asia, sub-Saharan Africa, and Latin America and the Caribbean.^3,16^ The data to determine the number needed to harm in the defined World Bank regions were calculated by clustering data from individual countries within their respective regions. In addition, we clustered 5-year episodes of SHS-associated death because of incomplete data on the number of individuals who died of SHS exposure in certain geographic areas. The SHSIs for various geographical areas were calculated with the same equation used to calculate the global SHSI.

### Statistical Analysis

All statistical analysis was performed with R Core Team 2019 (R Project for Statistical Computing). Because only descriptive statistics were calculated, no threshold of statistical significance was set.

## Results

### Baseline Characteristics of Individuals Who Smoked and Individuals Who Died of SHS Exposure Worldwide

**Figure 1.**
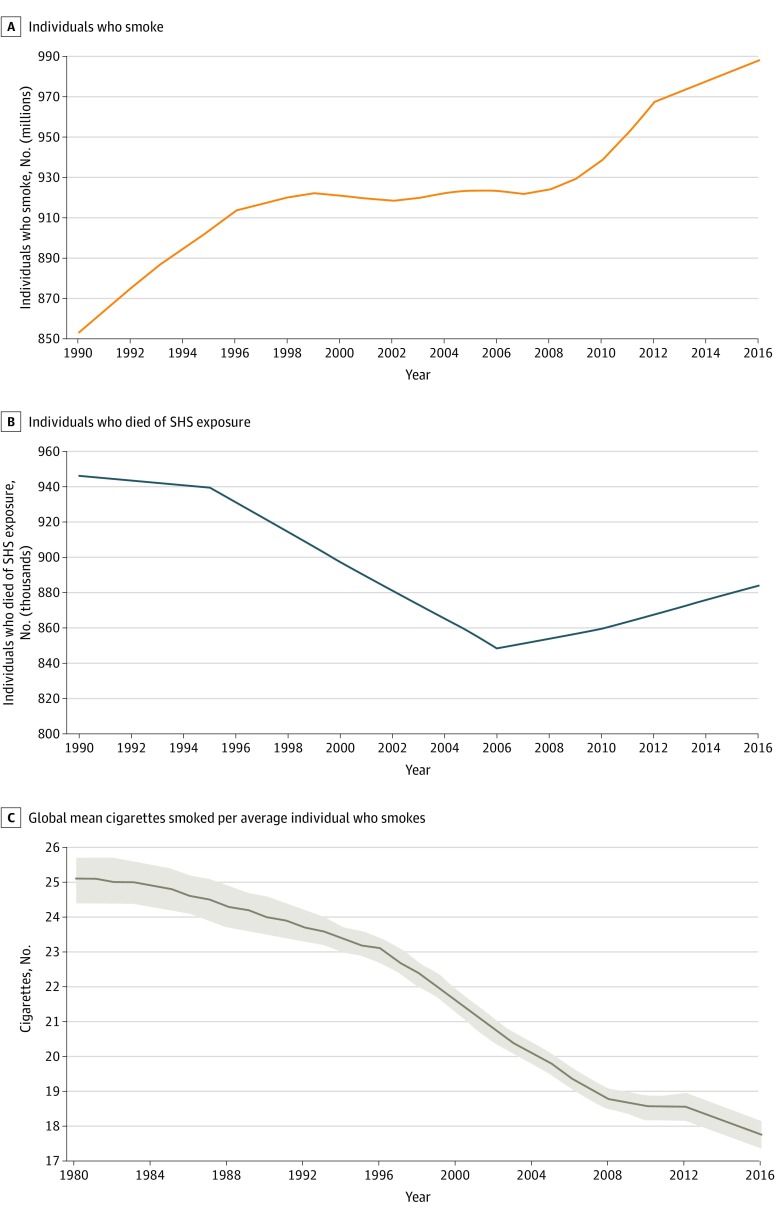
Absolute Numbers of Individuals Who Smoked and Who Died of Secondhand Smoke (SHS) Exposure, 1996 to 2016 C, Shaded areas represent the lower and upper bounds of the estimated mean cigarettes smoked per individual worldwide.

The absolute number of people who smoked between 1990 and 2016 has increased, mostly owing to the increase in smoking in low- to middle-income countries, especially China and India (Figure 1A). In 1990, 853 619 292 individuals smoked, and in 2016, 987 985 450 individuals smoked. The estimated absolute number of individuals who died of SHS exposure had decreased from 946 041 in 1990 to approximately 848 702 in 2006, but it started to gradually increase thereafter to 883 930 in 2016 (Figure 1B). This increase in the number of individuals who died of SHS exposure was mainly associated with the increase in deaths associated with SHS exposure in South Asia, East Asia, and the Pacific. The mean number of cigarettes consumed by an individual who smokes daily decreased from 25 to 18 cigarettes per day between 1990 and 2016 (Figure 1C). In addition, we reviewed the proportion of individuals who died of SHS exposure over time by age (Table 1). These data show a substantial reduction in the proportion of children younger than 5 years dying from SHS exposure from 272 176 (28.8% of total deaths of SHS exposure) in 1990 to 53 566 (6.1%) in 2016.

**Table 1.** The Distribution of Global Deaths Associated with SHS By Age Group

Year	Deaths associated with SHS, No.	Individuals who died of SHS exposure by age, No. (%)				
		<5 y	5-14 y	15-49 y	50-69 y	≥70 y
1990	946 041	272 176 (28.77)	10 785 (1.14)	61 871 (6.54)	225 725 (23.86)	375 578 (39.70)
1991	944 769	258 961 (27.41)	10 581 (1.12)	63 583 (6.73)	228 729 (24.21)	382 915 (40.53)
1992	943 497	245 781 (26.05)	10 473 (1.11)	65 196 (6.91)	231 723 (24.56)	390 325 (41.37)
1993	942 225	232 541 (24.68)	10 270 (1.09)	66 898 (7.10)	234 708 (24.91)	397 807 (42.22)
1994	940 952	219 336 (23.31)	10 068 (1.07)	68 595 (7.29)	237 684 (25.26)	405 174 (43.06)
1995	939 680	206 166 (21.94)	9961 (1.06)	70 288 (7.48)	240 746 (25.62)	412 613 (43.91)
1996	931 136	195 166 (20.96)	9591 (1.03)	70 580 (7.58)	240 792 (25.86)	415 100 (44.58)
1997	922 591	184 057 (19.95)	9226 (1.00)	70 947 (7.69)	240 796 (26.10)	417 565 (45.26)
1998	914 047	173 029 (18.93)	8866 (0.97)	71 296 (7.80)	240 760 (26.34)	420 096 (45.96)
1999	905 503	161 994 (17.89)	8421 (0.93)	71 625 (7.91)	240 864 (26.60)	422 598 (46.67)
2000	896 958	150 958 (16.83)	8073 (0.90)	71 936 (8.02)	240 833 (26.85)	425 158 (47.40)
2001	889 051	142 870 (16.07)	7735 (0.87)	71 480 (8.04)	240 399 (27.04)	426 656 (47.99)
2002	881 143	134 727 (15.29)	7402 (0.84)	70 932 (8.05)	239 935 (27.23)	428 147 (48.59)
2003	873 236	126 619 (14.50)	6986 (0.80)	70 470 (8.07)	239 441 (27.42)	429 719 (49.21)
2004	865 328	118 550 (13.70)	6663 (0.77)	70 005 (8.09)	239 004 (27.62)	431 193 (49.83)
2005	857 421	110 436 (12.88)	6259 (0.73)	69 451 (8.10)	238 535 (27.82)	432 740 (50.47)
2006	848 702	104 984 (12.37)	6026 (0.71)	69 424 (8.18)	235 685 (27.77)	432 583 (50.97)
2007	851 384	100 293 (11.78)	5875 (0.69)	69 558 (8.17)	236 940 (27.83)	438 718 (51.53)
2008	854 067	95 570 (11.19)	5722 (0.67)	69 692 (8.16)	238 199 (27.89)	444 884 (52.09)
2009	856 750	90 901 (10.61)	5655 (0.66)	69 739 (8.14)	239 462 (27.95)	450 993 (52.64)
2010	859 432	86 201 (10.03)	5500 (0.64)	69 872 (8.13)	240 727 (28.01)	457 132 (53.19)
2011	863 515	80 825 (9.36)	5267 (0.61)	69 772 (8.08)	245 238 (28.40)	462 412 (53.55)
2012	867 598	75 394 (8.69)	5119 (0.59)	69 755 (8.04)	249 781 (28.79)	467 635 (53.90)
2013	871 681	69 909 (8.02)	4969 (0.57)	69 647 (7.99)	254 357 (29.18)	472 887 (54.25)
2014	875 764	64 456 (7.36)	4729 (0.54)	69 536 (7.94)	258 876 (29.56)	478 080 (54.59)
2015	879 847	59 038 (6.71)	4575 (0.52)	69 508 (7.90)	263 426 (29.94)	483 388 (54.94)
2016	883 930	53 566 (6.06)	4420 (0.50)	69 389 (7.85)	267 919 (30.31)	488 637 (55.28)
Mean	892 824.61	141 277.93 (15.82)	7378.41 (0.83)	69 297.96 (7.76)	241 899.41 (27.09)	432 990.11 (48.50)
Median	881 143.00	126 619.00 (14.37)	6986.00 (0.79)	69 739.00 (7.91)	240 399.00 (27.28)	427 719.00 (48.77)
95% CI, No.	879 876.04-905 773.18	116 451.41-166 104.44	6567.48-8189.33	68 412.23-70 138.70	238 284.09-245 514.73	421 666.85-444 313.37

Abbreviation: SHS, secondhand smoke.

### The PYI

**Figure 2.**
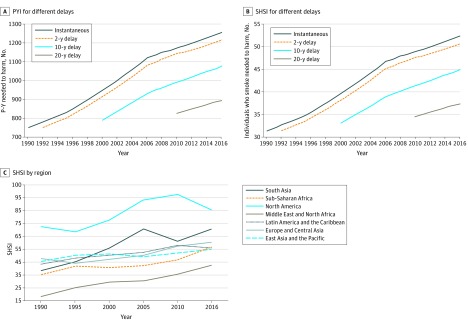
Global and Regional Secondhand Smoke Index (SHSI) and Pack-Year Index (PYI), 1996 to 2016 P-Y indicates pack-years.

The number of pack-years associated with the death of 1 individual who did not smoke (ie, the PYI) with no delay increased between 1990 and 2016 from 751.9 (95% CI, 736.3-770.7) pack-years to 1255.9 (95% CI, 1227.2-1284.4) pack-years (Figure 2A). Adjusting the delay between the number of individuals actively smoking and the number of individuals who died of SHS exposure only marginally changed the PYI. For example, associating the number of individuals who died of SHS exposure in 2016 with the number of pack-years smoked resulted in a minimal change in the PYI from 1255.9 (95% CI, 1227.2-1284.4) pack-years in 2016 to 1215.4 (95% CI, 1190.1-1245.8) pack-years in 2014. However, longer delays of 10 and 20 years for the number of individuals who died of SHS exposure in 2014 resulted in a change to 1077.2 (95% CI, 1054.8-1104.1) pack-years and 895.4 (95% CI, 876.7-917.7) pack-years, respectively.

### The SHSI

Worldwide, a gradual change in the number of individuals who smoked associated with the death of 1 individual who did not smoke was observed, decreasing from 31.3 (95% CI, 30.6-32.0) individuals who smoked in 1990 to 52.3 (95% CI, 51.2-53.5) individuals who smoked in 2016 (Figure 2B*)*. The mean number of cigarettes smoked per day in the same interval decreased from 24.0 (95% CI, 23.5-24.6) in 1990 to 17.8 (95% CI, 17.2-18.0) in 2016. Similar to the PYI, we used variable delays between SHS exposure and the occurrence of harm. For instance, associating the number of individuals who died of SHS exposure in 2016 with the number individuals who smoked in 2016 resulted in an SHSI of 52.3 (95% CI, 51.2-53.5) individuals who smoked, whereas associating the number of individuals who died of SHS in 2016 with the number of individuals who smoked in 2014 showed an SHSI of 50.6 (95% CI, 49.5-51.8) individuals who smoked. A further decrease in the number of individuals who smoked associated with the death of 1 individual exposed to SHS in 2016 was seen when increasing the delay to 10 years (44.9 [95% CI, 43.9-45.9] individuals who smoked) and 20 years (37.3 [95% CI, 36.5-38.2] individuals who smoked) (Figure 2B).

### Geographic Differences in the SHSI

The SHSI in different World Bank regions is presented in Figure 2C and Table 2. Because of incomplete data pertaining to individuals who died of SHS exposure in some regions, we presented the geographic SHSI data in 5-year clusters. The data showed substantial changes in the SHSI during the study period; the index was low in 1990 in South Asia (38.3 [95% CI, 37.5-39.3] individuals who smoked) and significantly improved in 2016 (70.6 [95% CI, 69.0-72.2] individuals who smoked). In Sub-Saharan Africa, the SHSI was low in 1990 (35.4 [95% CI, 34.6-36.2] individuals who smoked) and improved to 56.6 (95% CI, 55.3-57.9) individuals who smoked in 2016. In East Asia and the Pacific, the Middle East and North Africa, and Europe and Central Asia, an increase in the SHSI occurred from 1990 to 2016. In the Middle East and North Africa, 42.6 (95% CI, 41.6-43.5) individuals who smoked were associated with 1 individual who died of SHS exposure in 2016. In North America in 1990, the SHSI was already high and showed further improvement over time, increasing from 72.5 (95% CI, 71.0-74.3) individuals who smoked in 1990 to 85.7 (95% CI, 83.8-87.7) individuals who smoked in 2016.

**Table 2.** The Secondhand Smoke Index in Different Regions, 1990 to 2016

Year	Individuals Who Smoked Associated With the Death of 1 Individual Who Did Not, No.						
	South Asia	Sub-Saharan Africa	North America	Middle East and North Africa	Latin America and the Caribbean	Europe and Central Asia	East Asia and the Pacific
1990	38.31	35.35	72.49	18.40	43.45	47.74	45.57
1995	45.29	41.92	68.61	25.22	48.10	44.11	50.41
2000	55.81	40.80	77.84	29.45	50.39	47.33	51.34
2005	70.80	42.31	93.19	30.38	52.60	50.29	49.28
2010	61.13	46.68	97.62	35.63	58.00	57.28	52.52
2016	70.62	56.60	85.73	42.56	56.10	60.42	55.03

## Discussion

The major aim of this study was to provide a better understanding of the scale of the harm of SHS on individuals who do not smoke, with the goal of helping policy makers implement measures to protect those who do not smoke, especially children. Second, it is expected that creating quantitative measures, such as the SHSI and PYI, could increase awareness in the general public about the extent of damage associated with SHS.

Based on global data, we reported that 52.3 individuals who smoked for 24 years were associated with the death of 1 individual who did not smoke. Since 1990, the SHSI gradually increased from 31.3 individuals who smoked to 52.3 individuals who smoked in 2016. This change likely reflects the partial efficacy of antitobacco measures, including smoking prohibitions in bars and restaurants, to protect those who do not smoke from SHS harm. However, the SHSI of 52.3 individuals who smoked should be considered alarming, especially because a substantial proportion of those with SHS exposure–related deaths are children.

We observed that in regions where smoking bans have been widely adopted, such as North America, the SHSI was more favorable than in areas with minimal or no protection from SHS, such as the Middle East and North Africa. Therefore, the SHSI could become an instrument used by government and nongovernment organizations to determine the level of protection that intervention measures might offer to those who do not smoke. In addition, our data showed that the adoption of antitobacco legislation in specified regions was associated with a significant increase in the SHSI, suggesting that the interval SHSI could be used to measure the success of antitobacco legislation or campaigns.

The evaluation of antitobacco legislation and campaigns has shown that the 2 measures that are most associated with reductions in tobacco use are increasing taxes and discussing SHS exposure, especially among children.^17,18^ Whereas increasing taxation is a deterrent, the association of discussions regarding SHS exposure with reductions in tobacco use is more complex. Insight into the scale of the harm of SHS may provide legislators and policy makers with a foundation to implement measures to better protect those who do not smoke. To adjust for the different associations of heavy vs light smoking, we calculated the PYI based on the number of smoking-years and the mean number of cigarettes consumed daily for the respective geographic regions.

In 2018, the government of Flanders, Belgium, banned smoking in cars whenever children are present.^19^ This ban followed similar measures in countries such as the United Kingdom, and it has sparked discussion in other countries, such as the Netherlands, about adopting similar bans. Although the Netherlands is widely regarded as a progressive country, with among the best health care systems in the world, and is known for adopting novel social trends, the Dutch government weighed the civil liberty of citizens over the protection accorded to children during transportation. We believe that research such as ours could help influence public opinion and policy makers in favor of measures and legislation aimed at the protection of those who do not smoke, including children. Even for the nations with widely accepted smoking bans, such as the US and the UK, stricter control might further protect those exposed to SHS. Studies from Malaysia and Bangladesh^20^ have demonstrated that, in many countries, there is still a 40% level of exposure among children. Unfortunately, larger proportions of the population in countries such as China and India have started smoking, and the number of individuals who actively smoke is likely to increase further. Because many parts of the world are still being affected by tobacco use, we hope that associating harm directly with those who smoke will help to influence public opinion against SHS exposure and encourage governments to enforce stringent antitobacco legislation. We suggest that the SHSI may be used as a benchmark for measuring the effectiveness of protection against tobacco and, as such, help governments shape their antitobacco policies.

### Limitations

There are several limitations of the current study. The data collected from diverse world regions vary in quality and measure. The study presented here could inspire countries to better monitor the consequences of SHS in a quantitative manner, for instance by adopting and implementing the World Health Organization smoking assessment questionnaire.^15^ A scientifically robust approach to measuring SHS exposure would be to measure urinary cotinine excretion of individuals who do not smoke. Data from the Third National Health and Nutrition Examination Survey showed a detectable level of cotinine in 88% of adults who do not smoke, and numerous studies have validated questionnaire assessments of SHS exposure using cotinine measures.^21,22^ These studies have demonstrated that persons who were classified as having high levels of SHS exposure (often living with someone who smokes) on the basis of a survey had higher levels of serum, urinary, salivary, or hair biomarkers compared with those with low levels of exposure (often not living with anyone who smokes). Second, because the data sets we used only supplied the number of individuals who smoked in a given year and the number of individuals who died of SHS exposure, we needed to apply correction for the validity of the association. The commonest way of associating exposure to risk and occurrence of disease is to compare populations with and without the risk exposure and identify differences in the incidence of disease; this approach has been used in other studies, such as the National Health and Nutrition Examination Surveys. The approach of confirming long-term associations based on risk factors can be calculated as the Framingham Risk Score for 10 years or lifetime risk.^13^ However, studies supporting smoking bans have explicitly shown that the reduction in disease incidence largely occurs within 1 year and flattens out after 2 years.^2^ These data suggest that SHS affects those who do not smoke, including fetuses and children, within a short time span. For certain diseases related to SHS exposure, a long delay between exposure and the occurrence of disease is unquestionable, eg, in cases of lung cancer. However, of various causes of death related to SHS exposure, lung cancer plays only a minor role.^2^ To further investigate the association of variation in the delay between the number of individuals who died of SHS exposure in a given year and individuals who smoked in previous years, we calculated the PYI and the SHSI based on delays of 0, 2, 10, and 20 years. Even after the use of variable time intervals, the PYI and SHSI did not substantially change. Given that studies on the consequences of smoking bans show improvement for up to 2 years, it seems justifiable to allow a 2-year delay in the remaining calculations.

## Conclusions

Based on existing high-quality data sets retrieved from OWID, we calculated the mean number of pack-years needed to cause death in 1 individual who does not smoke through SHS exposure; this increased from 751.9 pack-years in 1990 to 1255.9 pack-years in 2016. In addition, we calculated the number of individuals who smoked for 24 years associated with the death of 1 individual who does not smoke; this increased from 31.3 in 1990 to 52.3 in 2016. Finally, we observed 3-fold to 4-fold fold differences in the SHSI among World Bank regions, reflecting large disparities in protective measures to shield those who do not smoke, especially children, from the harm of SHS exposure.

## Notes

Supplement. **eAppendix.** Interpolation and Extrapolation of the Available Data Sets
