# Supplementary material for: Estimated Worldwide Mortality Attributed to Secondhand Tobacco Smoke Exposure, 1990-2016
Source: JAMA Netw Open. 2020 Mar 17;3(3):e201177. doi: 10.1001/jamanetworkopen.2020.1177 (PMC7078760; doi:10.1001/jamanetworkopen.2020.1177)
Supplement: Supplement. — eAppendix. Interpolation and Extrapolation of the Available Data Sets [file jamanetwopen-3-e201177-s001.pdf]

# Supplementary Online Content

Yousuf H, Hofstra M, Tijssen J, et al. Estimated worldwide mortality attributed to secondhand tobacco smoke exposure, 1990-2016. *JAMA Netw Open*. 2020;3(3):e201177. doi:10.1001/jamanetworkopen.2020.1177

**eAppendix.** Interpolation and Extrapolation of the Available Data Sets

This supplementary material has been provided by the authors to give readers additional information about their work.

© 2020 Yousuf H et al. *JAMA Network Open.*

## **eAppendix.** Interpolation and Extrapolation of the Available Data Sets

We based the calculations on the impact of SHS on existing high quality datasets . The datasets taken from www.ourworldindata.org mostly contained data for the following years: 1990, 1995, 2000, 2005, 2006, 2010 and 2012 or 2016. In order to get a better overview of the data in the years missing in the dataset both linear inter- and extrapolation were conducted. The extrapolation was only necessary for the datasets that contained data until 2012.

Mathematically, linear interpolation can be presented as follows:

$$Y(\theta) = Y(n-1) + \frac{Y(n)-Y(n-1)}{X(n)-X(n-1)} * \theta$$

Where n is the nearest upper observation and n-1 the nearest lower observation, both with corresponding Y and X values. $\theta$ is the point of interest which lies between n-1 and n. This can be clarified with an example, in the table below the amount of smokers in an arbitrary country are presented in 1990 and 1995.

*Example table*

| **Year** | **Smokers** |
|---|---|
| 1990 | 120.000 |
| 1991 | *Y(1991)* |
| 1992 | *Y(1992)* |
| 1993 | *Y(1993)* |
| 1994 | *Y(1994)* |
| 1995 | 160.000 |

The amount of smokers (Y-values) for 1991 to 1994 are missing. Hence, linear interpolation is necessary to estimate the amount of smokers for these years.

© 2020 Yousuf H et al. *JAMA Network Open.*

Y(1991) can be estimated by filling in the formula as follows:

$$Y(1991) = Y(1990) + \frac{Y(1995) - Y(1990)}{X(1995) - X(1990)} * 1 = 120.000 + \frac{160.000 - 120.000}{5} * 1 = 128.000$$

In the case of Y(1991), $\theta$ is 1 since it one year later than the nearest lower X value with a corresponding observed y-value.

For the estimation of Y(1994) the formula is presented as follows:

$$Y(1994) = Y(1990) + \frac{Y(1995) - Y(1990)}{X(1995) - X(1990)} * 4 = 120.000 + \frac{160.000 - 120.000}{5} * 4 = 152.000$$

Now $\theta$ is equal to 4 since 1994 is 4 years later than 1990.

Linear extrapolation works in a similar way, mathematically it can be presented as follows:

$$Y(n + \theta) = Y(n) + \frac{Y(n) - Y(1)}{X(n) - X(1)} * \theta$$

With *n* being the observation corresponding to the most recent observed x and y value and n=1 being the oldest (in the case of time series) observation in the dataset with an observed x and y value. $\theta$ is the amount of time periods later than n.

The datasets with data on the amount of SHS-victims contained data until 2016, so linear extrapolation was not necessary. The datasets with data on the amount of smokers and daily cigarette consumption contained data until 2012 and thus linear extrapolation had to be conducted.

© 2020 Yousuf H et al. *JAMA Network Open.*
